# Unexplored Molecular Features of the *Entamoeba histolytica* RNA Lariat Debranching Enzyme Dbr^1^ Expression Profile

**DOI:** 10.3389/fcimb.2018.00228

**Published:** 2018-07-04

**Authors:** Jesús Valdés, Carlos Ortuño-Pineda, Odila Saucedo-Cárdenas, María S. Mendoza-Figueroa

**Affiliations:** ^1^Departamento de Bioquímica, Centro de Investigación y de Estudios Avanzados del Instituto Politécnico Nacional, Mexico City, Mexico; ^2^Unidad Académica de Ciencias Químico Biológicas, Universidad Autónoma de Guerrero, Chilpancingo, Mexico; ^3^Histología, Facultad de Medicina, Universidad Autónoma de Nuevo León, San Nicolás de los Garza, Mexico; ^4^División de Genética, Centro de Investigación Biomédica del Noreste, Instituto Mexicano del Seguro Social, Monterrey, Mexico; ^5^Departamento de Atención a la Salud, Universidad Autónoma Metropolitana-Xochimilco, Mexico City, Mexico

**Keywords:** *Entamoeba histolytica*, lariat, Dbr1, mRNA, splicing

## Abstract

The RNA lariat debranching enzyme (Dbr1) has different functions in RNA metabolism, such as hydrolyzing the 2′-5′ linkage in intron lariats, positively influencing Ty1 and HIV-1 retrotransposition, and modulating snRNP recycling during splicing reactions. It seems that Dbr1 is one of the major players in RNA turnover. It is remarkable that of all the studies carried out to date with Dbr1, to our knowledge, none of them have evaluated the expression profile of the endogenous *Dbr1* gene. In this work, we describe, for the first time, that *Entamoeba histolytica EhDbr1* mRNA has a very short half-life (less than 30 min) and encodes a very stable protein that is present until trophozoite cultures die. We also show that the EhDbr1 protein is present in the nuclear periphery on the cytoplasmic basal side, contrary to the localization of human Dbr1. Comparing these results with previous hypotheses and with results from different organisms suggests that *Dbr1* gene expression is finely tuned and conserved across eukaryotes. Experiments describing the aspects of *Dbr1* gene expression and *Dbr1* mRNA turnover as well as other functions of the protein need to be performed. Particularly, a special emphasis is needed on the protozoan parasite *E. histolytica*, the causative agent of amoebiasis, since even though it is a unicellular organism, it is an intron-rich eukaryote whose intron lariats seem to be open to avoid intron lariat accumulation and to process them in non-coding RNAs that might be involved in its virulence.

## Introduction

The spliceosome mediates intron removal and exon ligation of pre-mRNA through two consecutive trans-esterification reactions, resulting in a 2′-5′ linked lariat and in mRNA molecules (Padgett et al., 1984; Ruskin et al., 1984; Ruskin and Green, 1985; Konarska et al., 2006; Smith et al., 2009). Intron turnover is carried out by the RNA lariat debranching enzyme (Dbr1) that hydrolyzes the 2′-5′ linkage, opening the lariats (Ruskin and Green, 1985) either to be degraded or processed in non-coding RNAs (Ooi et al., 1998). After splicing, lariat RNA is present in two different post-splicing complexes, Intron Large (IL) and Intron Small (IS), but Dbr1 is not associated with either of them, indicating that Dbr1 association with lariat molecules is transitory (Yoshimoto et al., 2009; Garrey et al., 2014). Consistent with its function, the *Homo sapiens* Dbr1 (HsDbr1) in HeLa cells is localized in the nucleoplasm (Kataoka et al., 2013).

Dbr1 protein sequences from different organisms show high homology (Nam et al., 1997; Kim et al., 2000; Kataoka et al., 2013), suggesting that Dbr1 both in structure and in function is phylogenetically conserved. Dbr1 enzymes belong to the metallophosphoesterase (MPE) family (Koonin, 1994), showing a conserved N-terminal domain, a C-terminal domain (CTD) that does not show sequence similarity to any other class of proteins; a third domain between them, the lariat recognition loop (LRL) adjacent to the active site, is not present in other MPEs (Kim et al., 2000). As MPEs, Dbr1 enzymes use divalent metals for their activity (Arenas and Hurwitz, 1987), such as manganese in the case of the *Saccharomyces cerevisiae* Dbr1 (ScDbr1) (Khalid et al., 2005) or Fe^2+^, Zn^2+^, or Mn^2+^ in the case of *Entamoeba histolytica* Dbr1 (EhDbr1) (Clark et al., 2016; Ransey et al., 2017), suggesting a different requirement of metal cofactors.

To date, only the protozoan EhDbr1 has been crystallized (Montemayor et al., 2014; Clark et al., 2016; Ransey et al., 2017) from these structures, with the observation that a trinucleotide formed by the adenosine branch point flanked by 2′-5′ and 5′-3′ bonds is the minimal substrate for the debranching enzyme (Arenas and Hurwitz, 1987), Montemayor and coworkers proposed that the lariat molecule seems to be the determinant instead of a specific sequence for substrate recognition by Dbr1, allowing for the debranching of a diverse set of lariat RNA molecules (Montemayor et al., 2014). It is proposed that the hydrolysis of the debranching reaction is a SN2 mechanism via a trigonal bipyramidal pentacoordinate intermediate that results in an investment of the configuration, after which the 2′-O leaving group is protonated (Clark et al., 2016).

It has been observed that in *S. cerevisiae Dbr1* mutants that exhibit intron accumulation (Chapman and Boeke, 1991) and complementation with HsDbr1 (Kim et al., 2000), with the *Caenorhabditis elegans* Dbr1 (CeDbr1) (Nam et al., 1997), or with EhDbr1 (Montemayor et al., 2014), such a phenotype is rescued.

In the present work, we describe unstudied traits of the *E. histolytica* Dbr1 protein, whose structure has been determined recently (Montemayor et al., 2014). We found that the *EhDbr1* mRNA half-life is less than 30 min, but in contrast, it encodes a very stable protein that seems to be present until trophozoite cell cultures die. Additionally, we show that contrary to HsDbr1, EhDbr1 protein is present in the nuclear periphery on the cytoplasmic basal side, indicating that lariat opening in *E. histolytica* is carried out in a different cell compartment and suggesting that intron turnover in this organism might be regulated in a different way than humans. These data and data from other organisms suggest that *Dbr1* gene expression is finely tuned and conserved across eukaryotes. Thus, studies with an emphasis on *Dbr1* gene expression need to be performed. Particularly, in the protozoan parasite *E. histolytica*, EhDbr1 is possibly needed to open intron lariats that seem to be precursors of non-coding RNAs with unknown functions and is expressed from virulence genes (Mendoza-Figueroa et al. this issue).

## Materials and methods

### *Entamoeba histolytica* cell cultures

*E. histolytica* trophozoites from HMI: IMSS strain were axenically grown at 37°C in TYI-S-33 medium (Diamond et al., 1978) until they reached the exponential growth phase. Cells were harvested first by incubating them in an ice-water bath and then collected by centrifugation. Trophozoites were used immediately to extract total RNA or proteins by the TRIzol method (Invitrogen) according to the manufacturer's instructions.

### HeLa and MRC-5 cell cultures

Human cervical carcinoma (HeLa) cells were grown on coverslips inside Petri dishes and cultured in DMEM (Life Technologies). MRC-5 cells were grown in RPMI (Life Technologies). Both cultures were supplemented with 10% fetal bovine serum and were incubated at 37°C in a humid atmosphere of 5% CO_2_.

### RT-PCRs

Retrotranscription (RT) reactions were carried out with the M-MLV retrotranscriptase (Invitrogen) according to manufacturer's instruction. As template 10, 0.5, and 0.5 μg of total RNA were used in order to produce the *EhDbr1, 18S rRNA*, and *EhActin* cDNAs, respectively. Polymerase Chain Reactions (PCR) were carried out with 10% cDNA and the Taq DNA Polymerase (Invitrogen) according to manufacturer's instruction. Primers and PCR conditions are indicated in Table S1. As control in each experiment, PCR reactions with total RNA as template were carried out. Amplicons were analyzed in agarose gels and stained with ethidium bromide.

### *EhDbr1* mRNA half-life

**Figure 1:**
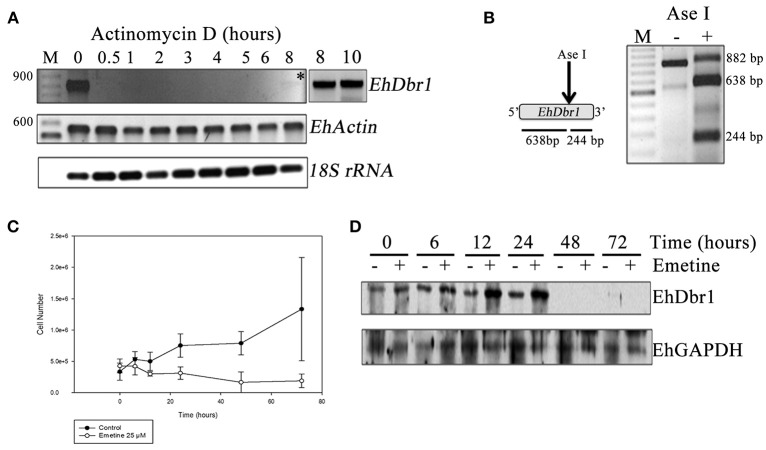
*EhDBR1* mRNA has low abundance, but the EhDbr1 protein has high stability. **(A)** Inhibition of RNA polymerase II transcription in *E. histolytica* trophozoites with Actinomycin D. *EhDbr1, EhActin*, and *18S rRNA* gene expression was evaluated to assess the *EhDbr1* mRNA half-life. Recuperation of *EhDbr1* expression is indicated with an asterisk; last two lanes in the left upper panel show PCR reactions of the 8 h sample, with additional (45 and 50) PCR amplification cycles. **(B)** Digestion of the EhDbr1 amplicon obtained in **(A)**, with the enzyme AseI as predicted from the restriction map. **(C)** *E. histolytica* trophozoite growth curve without or after 25μM emetine treatment. **(D)** EhDbr1 protein half-life measured by western blots from the previous trophozoite cultures.

Eighty percent confluent cell cultures of *E. histolytica* trophozoites were incubated in the presence of Actinomycin D to a final concentration of 1.3 nM (Ayala et al., 1990; Lopez-Camarillo et al., 2003) during the periods indicated in Figure 1A. Then, total RNA was extracted and *EhDbr1* gene expression was analyzed by RT-PCR. As control *EhActin* and *18S rRNA* gene expression were analyzed. The treatment was made by triplicate.

### EhDbr1 protein half-life

Eighty percent confluent cell cultures of *E. histolytica* trophozoites were incubated with the protein synthesis inhibitor emetine (Sigma-Aldrich) to a final concentration of 25 μM (Grollman, 1966) during the periods indicated in Figures 1C,D. After that, cells were counted (Figure 1C) and total proteins extracted by incubating cells in extraction buffer [10 mM HEPES-KOH, pH 7.2, 24 mM KCl, 10 mM MgCl_2_, 1 mM PMSF, 2 mM DTT, 1% NP-40, in the presence of the protease inhibitor cocktail “Complete” (Roche)] during 30 min with gentle agitation on ice. Samples were centrifuged at 6,000 × g 10 min at 4°C and proteins in the supernatant were quantitated with the DC Protein Assay Kit (Bio-Rad). Western blot assays were carried out in order to detect EhDbr1 and EhGAPDH proteins. The antibody Dbr1 (ThermoFisher Scientific) was used in a dilution 1:1,500 and the anti GAPDH (Santa Cruz Biotechnology) in a dilution 1: 10,000. The treatment was made by triplicate.

### Nuclear and cytoplasmic extracts

Nuclear and cytoplasmic extracts were obtained following the Dignam (Dignam et al., 1983) and Rio protocols (Rio et al., 2011). Both extracts were quantitated by the Bradford method and used for western blot assays in order to detect the EhDbr1 protein. Also western blots using the antibody EhCPADH (Garcia-Rivera et al., 1999), a gift from Dra. Esther Orozco, in a dilution 1: 40,000 and the antibody Tri-methyl-histone H3 (Lys4) (Cell Signaling) in a dilution 1: 1,000 were carried out as cytoplasmic and nuclear controls, respectively.

### EhDbr1 localization by confocal microscopy

*E. histolytica* trophozoites grown on coverslips were fixed and permeabilized with cold 100% methanol for 5 min and then blocked with 10% adult bovine serum for 1 h at room temperature. Cells were incubated overnight at 4°C with the first antibody Dbr1 (1:150) or with PBS (Phosphate Buffered Saline) as negative control. After several washes, samples were incubated with the Goat anti-Rabbit IgG rhodamine conjugated (1:100) (ThermoFischer Scientific). Nuclei were stained with 4′,6-Diamidino-2-Phenylindole (DAPI) and samples were observed through a confocal microscope (Carl Zeiss LSM 700) using the ZEN 2009 software. Observations were performed in approximately 20 optical sections from the top to the bottom of each sample.

## Results

### *EhDbr1* mRNA abundance is low, but the enzyme has high stability

Dbr1 cDNA has been isolated and has been used in complementation assays in different organisms (Nam et al., 1997; Kim et al., 2000). However, until now, knowledge about the *Dbr1* mRNA half-life has been missing. To determine the half-life of the *EhDbr1* mRNA, RNA polymerase II transcription was inhibited in *E. histolityca* trophozoites using Actinomycin D (Ayala et al., 1990; Lopez-Camarillo et al., 2003); after different incubation times with the drug, the total RNA was isolated, and the expression of *EhDbr1* was analyzed by RT-PCR. As Figure 1A shows, *EhDbr1* mRNA disappeared almost immediately after addition of the drug to the cultures. *EhDbr1* mRNA recuperated after 8 h.

Importantly, to amplify the *EhDbr1* transcript, 10 μg of total RNA was needed in the RT reaction, and extra PCR cycles were employed to better visualize the product at 8 h of Actynomycin D treatment (Figure 1A, lanes 8 and 10 to the right of the main panel), suggesting a low level of EhDbr1 transcripts. This result is consistent with data from *Arabidopsis thaliana*, where amplification of the *AtDbr1* transcript needed 40 PCR cycles compared to 24 for *AhTUB2*, indicating a low *AtDbr1* mRNA abundance (Wang et al., 2004).

The identity of the PCR product was corroborated by restriction analysis of the amplicon (Figure 1B). *EhActin* gene expression was analyzed to control RNA polymerase II inhibition and, the results were consistent with previous work (Lopez-Camarillo et al., 2003); its transcription was not inhibited (half-life >12 h). The expression of the *18S rRNA* was analyzed as a loading control and to control the resistance of RNA polymerase I to Actinomycin D (Oakes et al., 1993).

The EhDbr1 protein half-life was evaluated by inhibition of protein synthesis of *E. histolytica* trophozoites with emetine (Grollman, 1966). After incubation with the drug, the total protein was extracted and analyzed by western blots to detect the enzyme as well as to detect EhGAPDH as a loading control. In Figure 1C, the growth curve of *E. histolytica* trophozoites after emetine treatment is shown. Consistent with previous findings (Grollman, 1966), trophozoite growth was inhibited by the drug. Notably, EhDbr1 synthesis was inhibited only after 24 h of treatment (Figure 1D); however, protein disappearance coincided with the near absence of cells, suggesting that EhDbr1 disappearance is due to cell death instead of synthesis inhibition. This result indicates that EhDbr1 is present in the cells until trophozoites cultures die. It is interesting to note that the EhDbr1 signal disappears after 48 h, even in the absence of emetine. This result most likely occurs because starting at this time point, cell cultures are over 70% lysed and because at this time point EhDbr1 could be degraded faster than EhGAPDH.

### EhDbr1 localization

**Figure 2:**
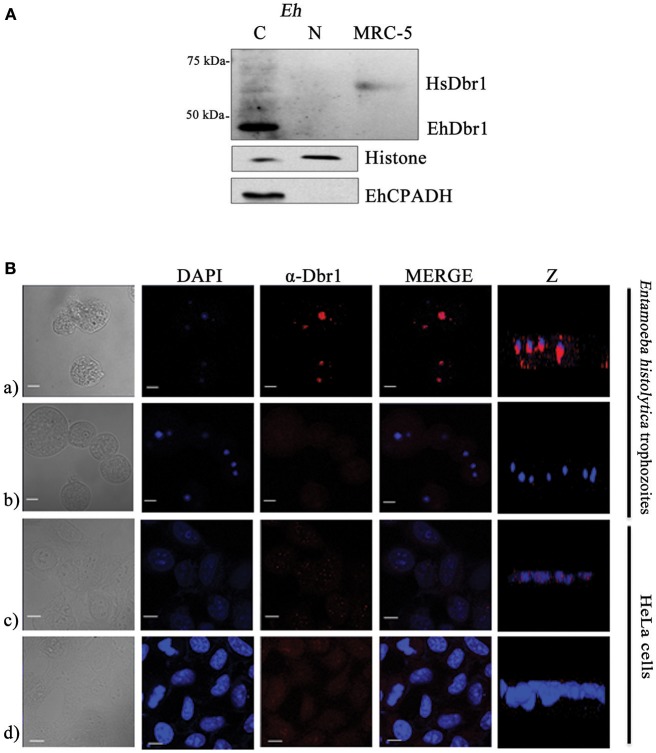
EhDbr1 cellular localization. **(A)** EhDbr1 protein localization was evaluated by cytoplasmic and nuclear fractionation of *E. histolytica* trophozoites. A sample of human (MRC-5 cells) proteins was included in the western blot. EhDbr1 is 42 kDa, and HsDbr1 is 61 kDa. Histone and EhCPADH were analyzed as cytoplasmic and nuclear indicators, respectively. **(B)** Apparently, EhDbr1 localizes in the cytoplasmic-basal nuclear periphery. (a,c) confocal immunofluorescence of *E. histolytica* and HeLa cells, respectively, using the anti-Dbr1 as the primary antibody and goat anti-rabbit IgG rhodamine conjugated secondary antibody (red). (b,d) same as above, without the primary antibody. Nuclei were stained with DAPI (blue). Z plane: lateral image of cells. Bar scale = 10 μm.

Consistent with its function, the HsDbr1 protein is mainly localized in the nucleus (Figure 2), but in certain circumstances, it has been observed in the cytoplasm (Kataoka et al., 2013). We set to determine EhDbr1 localization in *E. histolytica* trophozoites. First, nuclear- and cytoplasmic-enriched fractions were obtained, and EhDbr1 was detected by western blot. Unexpectedly, as seen in Figure 2A, the EhDbr1 enzyme was localized mainly in the cytoplasm and was nearly absent in the nucleus. As controls of nuclear and cytoplasmic fractionation, histones and the EhCPADH complex (Garcia-Rivera et al., 1999), respectively, were evaluated by western blot, confirming appropriate fractionation.

The Z planes of confocal images suggest that EhDbr1 is in the nuclear periphery on the cytoplasmic side (Figure 2Ba), toward the basal-substrate region. Taken together, these results suggest that EhDbr1 might be associated with the nuclear envelope on the cytoplasmic basal side of the *E. histolytica* trophozoites.

## Discussion

One of the natural products of pre-mRNA splicing are intron lariat molecules, whose functions are less characterized compared to mRNA. It seems that independent of its function, the 2′-5′ phosphodiester linkage in the intron lariats needs to be opened by the Dbr1 enzyme to process the lariats. It is remarkable that of all the studies carried out to date with Dbr1, to our knowledge, none of them have evaluated the expression profile of the endogenous *Dbr1* gene.

*EhDbr1* mRNA appears to have a half-life of less than 30 min, indicating a low abundance of the transcript in the cell under steady state conditions, possibly due to low *EhDbr1* gene transcription, which is consistent with the recuperation of transcription after 8 h of Actinomycin D treatment. Then, once translated, its degradation rate might be increased, resulting in a low accumulation of the *EhDbr1* transcript. In this scenario, the resultant protein should have high stability, such that high quantities of mRNA are not required. In *A. thaliana*, Wang and coworkers observed that the accumulation level of the *AtDbr1* transcript was low (Wang et al., 2004). As in this work, the authors needed a high number of PCR cycles to amplify the *AtDbr1* mRNA. Furthermore, because *S. cerevisiae, C. elegans* and *A. thaliana* contain a single copy of the *Dbr1* gene and because *H. sapiens* contain, at most, two copies (Kim et al., 2000; Wang et al., 2004), it has been proposed that *HsDbr1* mRNA abundance should be extremely low and that the encoded protein should have a high specific activity, perhaps being very stable (Kim et al., 2000). In *E. histolytica*, the *EhDbr1* gene copy number has not yet been determined, but we observed that the EhDbr1 protein is very stable.

First, low *Dbr1* mRNA levels in *E. histolytica*, as in *A. thaliana*, suggest that their RNA turnover is fast. Particularly, in amoeba, the mRNA half-life is influenced by the length of the 3′ end poly(A) tail, modified by stress conditions (Lopez-Camarillo et al., 2003, 2014). The poly(A) tail length of *EhDbr1* mRNA is unknown, but it will be important to determine this signature in the transcript to know if it is a determinant for the low accumulation observed. We do not know if other *E. histolytica* genes carry out a similar rate of RNA turnover.

Second, compared with the forward K_M_ values of constitutive glycolytic amoebic enzymes such as hexokinase (~33 μM for glucose), GAPDH (~33 μM for glyceraldehyde-3-phosphate) or the fructose bisphosphate aldolases that shows the highest affinities for their substrate Fru(1,6)P_2_ (~4 μM) (Saavedra et al., 2005), the K_M_ value for Dbr1 is 0.2–0.5 μM for short-branched RNAs (Garrey et al., 2014; Clark et al., 2016). These K_M_ values suggest that debranching enzymes have a high specific activity, as previously proposed (Kim et al., 2000), with turnover rates of ~3 s^−1^ (Clark et al., 2016). This activity is enhanced by Dnr1, a Dbr1 homolog protein, that directly interacts with the lariat (Garrey et al., 2014).

Thus, all of these data support the idea that *EhDbr1* gene expression regulation seems to be finely tuned and conserved across eukaryotes, such that cells synthesize few mRNA molecules that are translated into robust Dbr1 enzymes, constituting one of the key (and probably the most critical) factors that regulate intron lariat turnover.

Surprisingly, the EhDbr1 enzyme is present until trophozoite cultures die, suggesting that the half-life of the EhDbr1 enzyme may be longer than the trophozoite cell doubling time. We suspect that the longer half-life of this enzyme could be due to the absence of additional ubiquitination sites present in HsDbr1 (Figure S1), although this possibility remains to be tested. However, we can envisage the need of a robust EhDbr1 since, even though *E. histolytica* is a unicellular organism, it is an intron-rich eukaryote (Hon et al., 2013); thus, amoebic intron lariats need to be immediately open to degrade them, avoiding lariat accumulation, or to process them in ncRNAs, such as circular RNA molecules (Mendoza-Figueroa et al. this issue).

Because of the difference in the localization pattern between HsDbr1 and EhDbr1 shown by western blot analysis, we hypothesized that EhDbr1 has a weak nuclear localization signal (NLS), allowing its passage to the nucleus in minute quantities. The EhDbr1 protein sequence alignments with the bipartite NLS (VPLKRLSDEHEPEQRKKIKRRNQAIYAAVDDDDDDAA) of the HsDbr1 C-terminal domain (HsNLS), constituted by 37 amino acid residues (Kataoka et al., 2013) and the three portions (EhNLS1, 2, and 3) of the *E. histolytica* EhNCABP166 tripartite NLS (Uribe et al., 2012), showed that the single EhDbr1 NLS does not match perfectly to any of the NLS tested (Figure S2), supporting the notion of a weak NLS in EhDbr1 compared with the bipartite HsNLS and the tripartite EhNLS. This situation prevented the EhDbr1 from entering the nucleus as efficiently as HsDbr1 does. We do not discard the possibility that in other growth phases, EhDbr1 could be present abundantly inside the nucleus. These possibilities need further evaluation. EhDbr1 localization was analyzed by confocal microscopy. For comparison, nuclear speckles localization of the HsDbr1 in HeLa cells was included (Kataoka et al., 2013). Our results suggest that the EhDbr1 is localized on the cytoplasmic side of the nuclear periphery, appearing concentrated on the basal (toward the substrate) region. Taken together, these results suggest that the EhDbr1 might be associated with the nuclear envelope on the cytoplasmic side of the *E. histolytica* trophozoites, arguing for an inefficient NLS C-terminal EhDbr1 and allowing for minute amounts of nuclear EhDbr1 to be transported. It is also possible that in the growth phase analyzed, EhDbr1 mainly rests on the cytoplasmic side, while in other growth phases, it could be mainly nuclear. These hypotheses need to be evaluated.

The main function attributed to Dbr1 is to hydrolyze the 2′-5′ linkage in RNA lariats (Ruskin and Green, 1985); however, other functions have been associated with Dbr1. For example, the retrotransposon Ty1 replicates by retrotranscription, and the resultant cDNA then is then integrated into the host genome. In Δ*dbr1 S. cerevisiae* cells, Ty1 cDNA accumulates (Chapman and Boeke, 1991; Salem et al., 2003), suggesting that ScDbr1 is involved in retrotranscription or cDNA stability (Salem et al., 2003). Experiments carried out by Salem and coworkers ruled out the possibility that the Ty1 cDNA had 2′-5′ linkages at the 5′ or 3′ ends since it integrates into the genome in the Δ*dbr1* cells. Additionally, Pratico and Silverman demonstrated that a 2′-5′-branched RNA is not a retrotransposition intermediate, suggesting that Dbr1 binds RNA only to stabilize it or that it carries other functions different from the 2′-5′ bond cleavage during the Ty1 retrotransposition (Pratico and Silverman, 2007). To gain more insights into the role of Dbr1 in Ty1 retrotransposition, it will be important to determine if Dbr1 associates with Ty1 RNA directly in the nucleus or in the retrosomes, the cytoplasmic foci where the virus-like particles are assembled (Pachulska-Wieczorek et al., 2016). Moreover, HIV-1 is a retrovirus resembling retrotransposons. It seems that HIV-1 initiates its cDNA synthesis at the cytoplasm and then completes it in the nucleus or in the perinuclear region. The initial synthesis does not depend on HsDbr1, but its completion does. Currently, it is unknown if a lariat-like structure with a 2′-5′ bond is formed in the HIV-1 RNA such that HsDbr1 needs to open it (Galvis et al., 2014).

Recently, Han and coworkers showed that HsDbr1 modulates snRNP recycling, allowing the enzymes to dissociate from post-catalytic complexes and to be available to form new spliceosomes complexes. If snRNP availability is compromised, the resulting effect is a change in the alternative splicing pattern that leads to tumorigenesis (Han et al., 2017).

To date, the role of Dbr1 in the cytoplasm is not clear; however, from Ty1 and HIV-1 data, it seems that Dbr1 might have a function different from that of 2′-5′ bond cleavage. EhDbr1, as well as other Dbr1 enzymes, may have a conserved cytoplasmic function not described thus far.

## Conclusion and future directions

In this work, we describe that *EhDbr1* mRNA has a very short half-life (less than 30 min) and encodes a very stable protein that is present until trophozoites cultures die. Moreover, in a steady state, *E. histolytica* trophozoite EhDbr1 seems to be localized in the nuclear periphery, probably because of the lack of a well-defined nuclear localization signal. These data and data from other organisms suggest that Dbr1 gene expression is finely tuned and conserved across eukaryotes. Experiments that describe the aspects of *EhDbr1* gene transcription and *EhDbr1* mRNA turnover, as well as those describing other functions of the protein both in the nucleus and cytosol, need to be performed.

## Author contributions

MM-F conceived, designed, acquired data, carried out the experiments, analyzed data and drafted the manuscript; CO-P participated in the design of the study and drafted the manuscript; OS-C drafted the manuscript; JV conceived and designed the study, analyzed data and drafted the manuscript. All authors read and approved the final manuscript.

### Conflict of interest statement

The authors declare that the research was conducted in the absence of any commercial or financial relationships that could be construed as a potential conflict of interest.
