# Supplementary figures and images for: Unexplored Molecular Features of the Entamoeba histolytica RNA Lariat Debranching Enzyme Dbr1 Expression Profile
Source: Front Cell Infect Microbiol. 2018 Jul 4;8:228. doi: 10.3389/fcimb.2018.00228 (PMC6039765; doi:10.3389/fcimb.2018.00228)

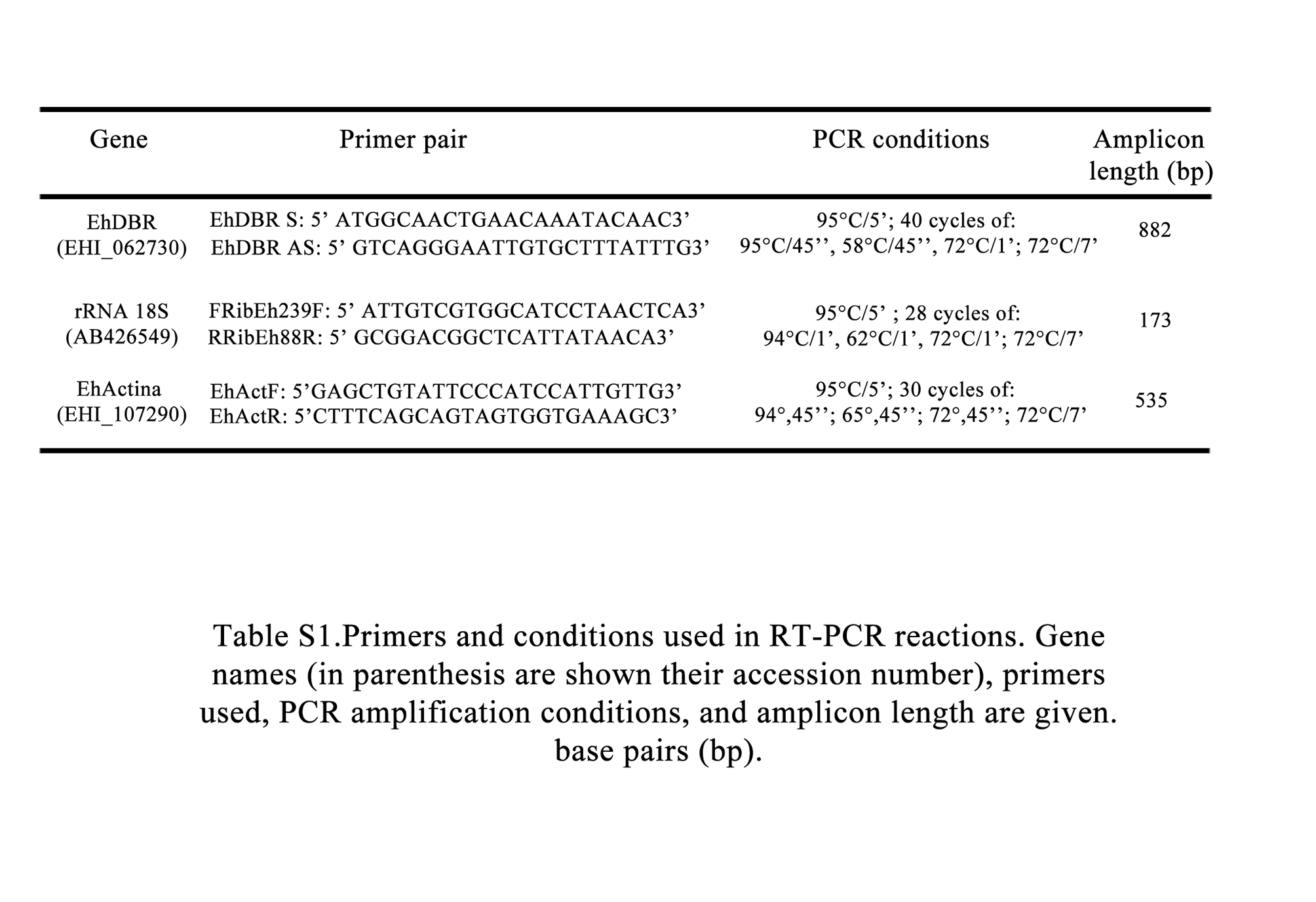

Supplement: Supplementary file 1 [file Image_1.TIF]

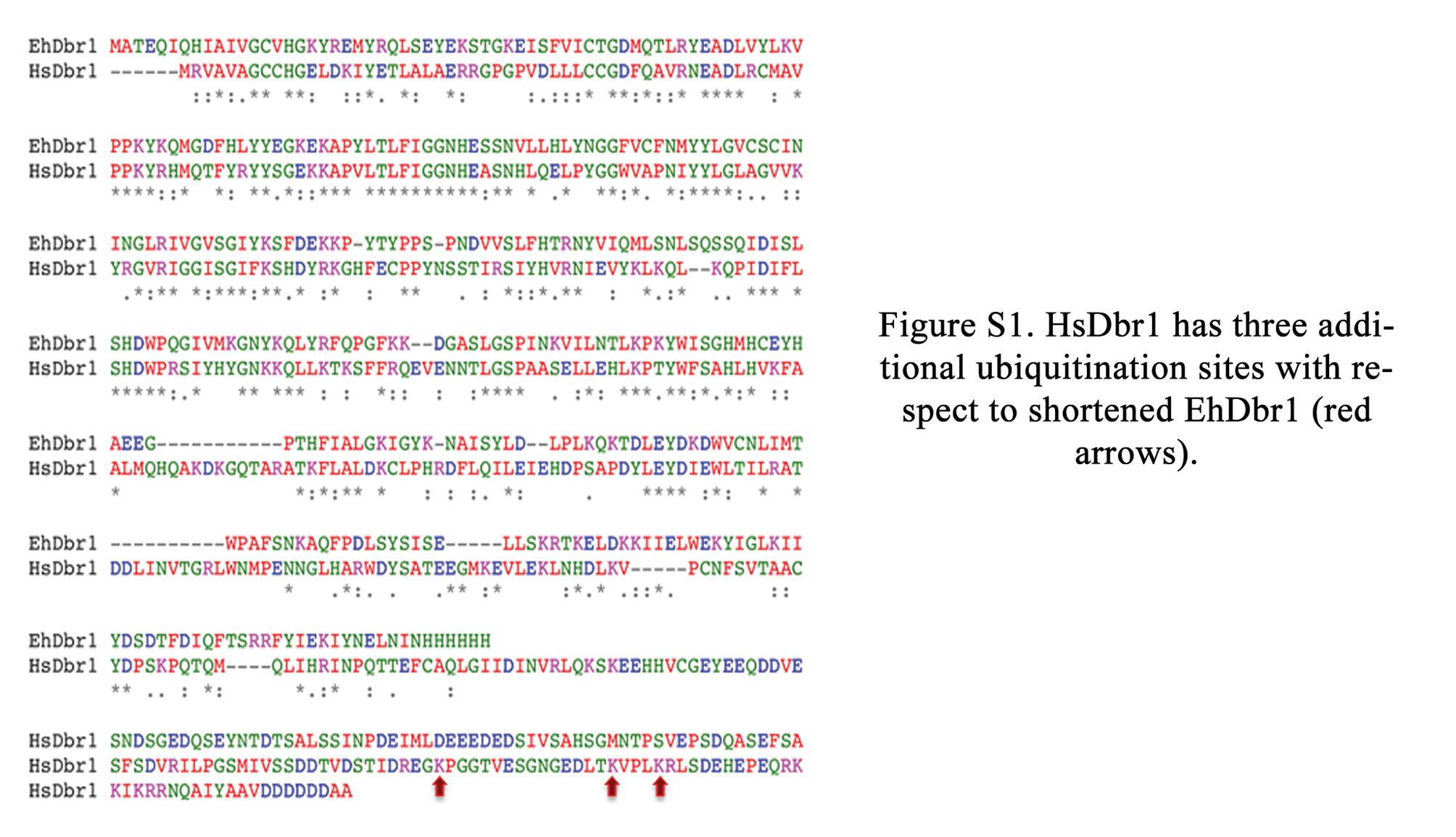

Supplement: Supplementary file 2 [file Image_2.TIF]

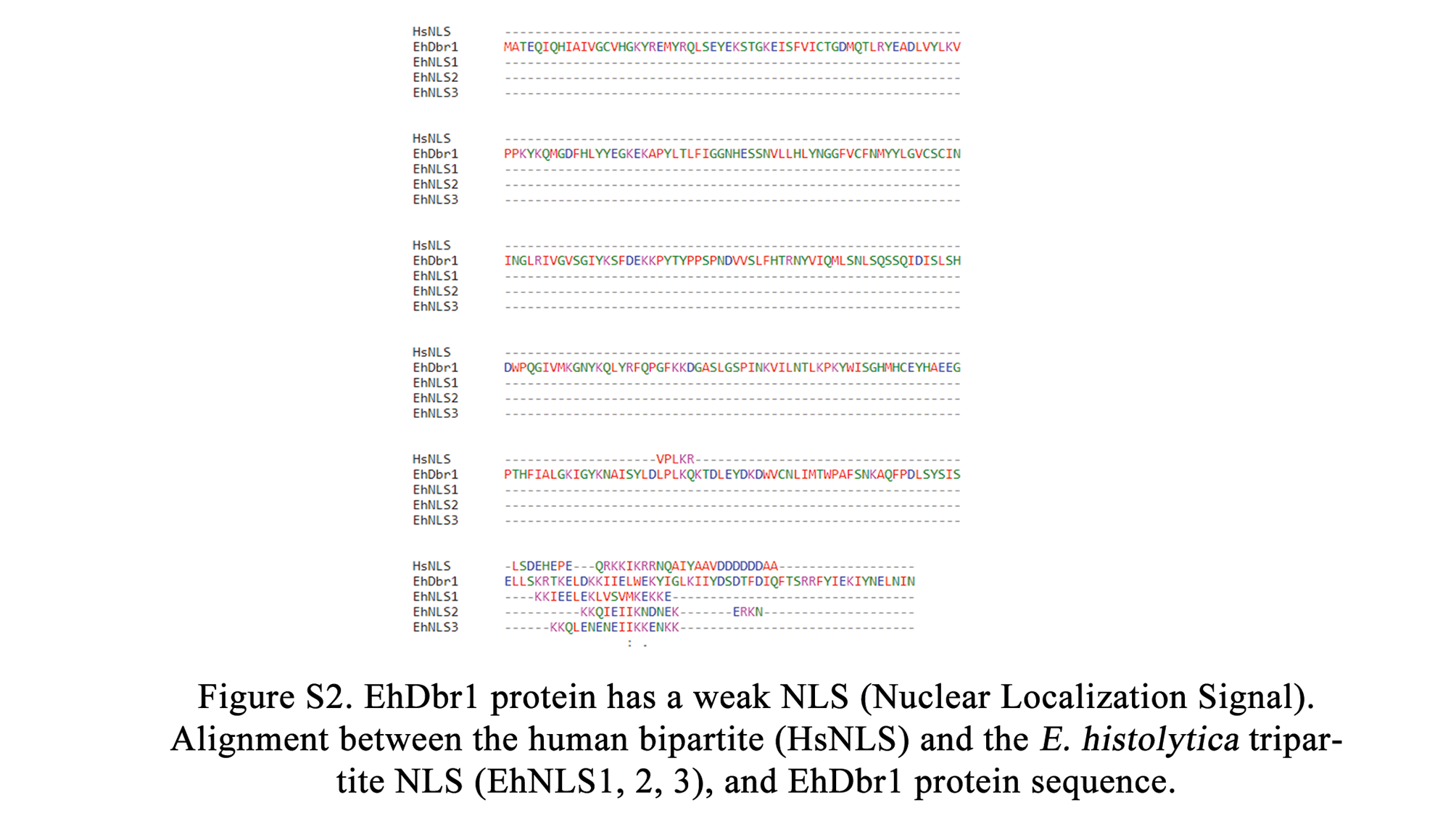

Supplement: Supplementary file 3 [file Image_3.TIF]
